# Impact of respiratory motion on lung dose during total marrow irradiation

**DOI:** 10.3389/fonc.2022.924961

**Published:** 2022-10-18

**Authors:** Ayse Gulbin Kavak, Murat Surucu, Kang-Hyun Ahn, Erik Pearson, Bulent Aydogan

**Affiliations:** ^1^ Department of Radiation Oncology, Faculty of Medicine, Gaziantep University, Gaziantep, Turkey; ^2^ Department of Radiation Oncology, Stanford University School of Medicine, Stanford, CA, United States; ^3^ Department of Radiation and Cellular Oncology, University of Chicago Pritzker School of Medicine, Chicago, IL, United States; ^4^ Department of Radiation Oncology, University of Illinois at Chicago Medical Center, Chicago, IL, United States

**Keywords:** organ motion, dose delivery, breathing motion, TMI, total marrow irradiation

## Abstract

We evaluated the impact of respiratory motion on the lung dose during linac-based intensity-modulated total marrow irradiation (IMTMI) using two different approaches: (1) measurement of doses within the lungs of an anthropomorphic phantom using thermoluminescent detectors (TLDs) and (2) treatment delivery measurements using ArcCHECK where gamma passing rates (GPRs) and the mean lung doses were calculated and compared with and without motion. In the first approach, respiratory motions were simulated using a programmable motion platform by using typical published peak-to-peak motion amplitudes of 5, 8, and 12 mm in the craniocaudal (CC) direction, denoted here as M1, M2, and M3, respectively, with 2 mm in both anteroposterior (AP) and lateral (LAT) directions. TLDs were placed in five selected locations in the lungs of a RANDO phantom. Average TLD measurements obtained with motion were normalized to those obtained with static phantom delivery. The mean dose ratios were 1.01 (0.98–1.03), 1.04 (1.01–1.09), and 1.08 (1.04–1.12) for respiratory motions M1, M2, and M3, respectively. To determine the impact of directional respiratory motion, we repeated the experiment with 5-, 8-, and 12-mm motion in the CC direction only. The differences in average TLD doses were less than 1% when compared with the M1, M2, and M3 motions indicating a minimal impact from CC motion on lung dose during IMTMI. In the second experimental approach, we evaluated extreme respiratory motion 15 mm excursion in only the CC direction. We placed an ArcCHECK device on a commercial motion platform and delivered the clinical IMTMI plans of five patients. We compared, with and without motion, the dose volume histograms (DVHs) and mean lung dose calculated with the ArcCHECK-3DVH tool as well as GPR with 3%, 5%, and 10% dose agreements and a 3-mm constant distance to agreement (DTA). GPR differed by 11.1 ± 2.1%, 3.8 ± 1.5%, and 0.1 ± 0.2% with dose agreement criteria of 3%, 5%, and 10%, respectively. This indicates that respiratory motion impacts dose distribution in small and isolated parts of the lungs. More importantly, the impact of respiratory motion on the mean lung dose, a critical indicator for toxicity in IMTMI, was not statistically significant (*p* > 0.05) based on the Student’s *t*-test. We conclude that most patients treated with IMTMI will have negligible dose uncertainty due to respiratory motion. This is particularly reassuring as lung toxicity is the main concern for future IMTMI dose escalation studies.

## Introduction

Changes in the patient anatomy are one of the largest contributors to uncertainties in dose delivery for radiation therapy. Within a single treatment delivery, i.e., intra-fraction, these changes are typically from organ motion related to physiological processes, such as digestion, cardiac motion, and respiration, with the latter typically being the most significant for treatments in the thorax. In the case of total marrow irradiation (TMI), the dose to the lung, as a critical organ at risk (OAR), is often a limiting factor. However, the impact of respiratory motion on the dose uncertainty in the lung has not been previously reported for TMI.

Total body irradiation (TBI) is an integral component of conditioning regimens prior to hematopoietic stem cell transplants. It performs two critical functions, eradicating the malignant cells escaping chemotherapy and immunosuppression to prevent the rejection of donor marrow or hematopoietic cells. Over the last two decades, the use of TBI has been steadily declining due mainly to concerns about toxicities, while alternative drug-based approaches are fast becoming the standard of care for the treatment of hematological malignancies (1–5). Various acute and chronic radiation toxicities reduce the quality of life for patients treated with TBI. Acute effects include temporary hair loss, nausea, vomiting, diarrhea, decreased blood cell count, mouth sores, and skin irritation. Among the chronic effects of TBI are cataracts, infertility, secondary malignancies, and decreased and delayed growth and development in children (2, 3). Toxicities induced by TBI inclusive conditioning regimens, such as pneumonitis, can be life-threatening (6–9). Several studies reported interstitial pneumonitis rates ranging from 6% to 30% with TBI (10–12). Della Volpe et al. (13) reported, in a retrospective study, an increase in lethal lung complications from 3.8% to 19.2% when the lung dose exceeded a threshold of 9.4 Gy. Furthermore, TBI dose escalation studies have failed due to increased fatal complications (8, 9) and are deemed impossible with current treatment techniques.

TMI has been introduced to replace TBI with the aim of reducing toxicity and enhancing the therapeutic ratio (14–16). The main advantage of TMI is the ability to focus radiation on targets and reduce radiation dose to organs at risk (OARs), particularly to the lungs, the dose-limiting organ (6, 17). TMI targets the entirety of the skeletal structure; consequently, most OARs are in close proximity to one or more target structures (18, 19). The lung can be particularly challenging to spare as it is tightly wrapped within the rib cage, a treatment target itself. It has been shown that linac-based intensity-modulated total marrow irradiation (IMTMI) and volumetric arc radiotherapy (VMAT-TMI) can reduce OAR dose by 29%–65% when compared with TBI (17, 20, 21). Similar results were also reported using the helical TMI technique (16).

Several clinical studies have established the clinical feasibility and tolerability of TMI in patients with advanced diseases as part of a conditioning regimen prior to allogeneic stem cell transplantation (22–24). TMI provides a potentially practice-changing RT technique that may allow dose escalation, better dose homogeneity, and lower toxicity. This may be expected to improve upon the current standard of care in the treatment of hematological malignancies and improve outcomes (19). Further studies to investigate technical and dosimetric challenges such as organ motion are imperative to limit toxicity and allow safe dose escalation, which is of great interest especially in the treatment of patients with advanced diseases.

It was suggested that the dose heterogeneity in both the PTV and surrounding healthy tissue increases with increasing respiratory motion amplitude (25). Most of our knowledge regarding lung motion comes from the studies that evaluated either a single point in tumor or internal markers using imaging or external surrogates with devices such as Real-Time Position Management System (RPM, Varian Medical Systems, Palo Alto, CA) (26). A point in the lung may exhibit large displacements due to respiratory motion, which results in significant geometric and dosimetric uncertainties (25–28). Knybel et al. (29) reported average motion amplitude changes to be 6.0 ± 2.2 mm and Liu et al. (30) reported that only 10.8% of the patients experienced tumor motion more than 10 mm. Seppenwoolde et al. (31) reported that the largest tumor motion was 12 ± 2 mm in the CC direction and 2 ± 1 mm in both the anteriorposterior (AP) and lateral (LAT) directions.

The steep dose gradients possible with IMRT enable better target conformity and healthy tissue sparing, especially for irregularly shaped concave target volumes. However, the sharp dose gradient can potentially lead to dose uncertainty due to imperfections in patient positioning, immobilization, and organ motion (32–34). The goal of this study is to evaluate the impact of respiratory motion on the lung dose during IMTMI.

## Materials and methods

### Treatment planning

We used the anthropomorphic RANDO phantom (The Phantom Laboratory, Salem, NY) for treatment planning and dose measurement. The RANDO phantom was scanned with a 3-mm slice thickness using a Picker PQ 5000 CT scanner (Philips Medical Systems, Cleveland, OH). The entire skeletal structure was contoured and expanded using a 3-mm isotropic margin to construct the PTV. IMTMI planning followed the technique described previously (4, 14, 15) using the Eclipse treatment planning system (Varian™ Medical Systems, Palo Alto, CA).

The contoured OARs included the following: lenses of the eyes, brain, oral cavity, lungs, liver, kidneys, heart, and small intestine. Each TMI plan had three sub-plans: one for the head and neck, one for the chest, and one for the pelvic region. PTVs in the head and neck sub-plan included the cranium, mandible, and cervical vertebral bodies (C1 to C7). The chest sub-plan included the sternum, ribs, and thoracic vertebral bodies (T1 to T12) and the pelvis sub-plan included the os coxae, femoral head, lumbar vertebral bodies (L1 to L5), and the upper half of the femur. The total prescribed IMTMI dose was 12 Gy. Nine equally spaced 6-MV IMRT beams were created for each sub-plan and optimized to deliver the prescription dose to provide 95% PTV coverage. In order to improve the homogeneity in the junction areas, the chest sub-plan was optimized first and then used as the base plan for both the pelvis and head and neck sub-plans. Although we used only the chest sub-plan in this study, a full IMTMI plan was generated to account for dose from abutting fields and to simulate the IMTMI treatment and actual dose a patient would receive in the clinic. Retrospective patient data in this study were obtained from an IRB-approved clinical trial.

### TLD sensitivities

We used thermoluminescent detectors (TLD-100) with a cross-section of 3 mm × 3 mm and a thickness of 0.9 mm. All TLDs were annealed before each exposure in a high-temperature Fisher Scientific Isotemp Oven (Fisher Scientific, Pittsburgh, PA) for 1 h at 400°C and 18 h at 80°C to decrease residual signals. After each exposure, the TLDs were stored at room temperature for 16 h prior to read out. A Harshaw 3500 TLD reader (Thermo Electron Corp., Santa Fe, NM) was used for TLD reading. TLD sensitivities were obtained using three independent exposures to a uniform dose of 0.85 Gy from a 6-MV beam under full scatter conditions, with a field size of 10 × 10 cm^2^, a source-to-axis distance of 100 cm, and at a depth of 10 cm in solid water. TLDs were examined according to the protocol defined by Reft et al. (35). The standard deviations of the calibration factors defined uncertainties in individual TLD sensitivities.

### Radiation measurement

We modified the plugs that were provided with the RANDO phantom to make enough room for three TLDs while keeping them securely in place to eliminate positioning uncertainty. We selected five points for measurement using the calculated dose distributions to be representative of doses ranging from low to high within the lungs. All points were at least 2 cm deep in the body and 5 cm away from the edge of the phantom to avoid any potential dosimetric error. Three TLD-100 chips were placed in each of the five predetermined locations in the lungs of the RANDO phantom. Each experiment was repeated three times to reduce measurement uncertainty for each simulated respiratory motion.

**Figure 1:**
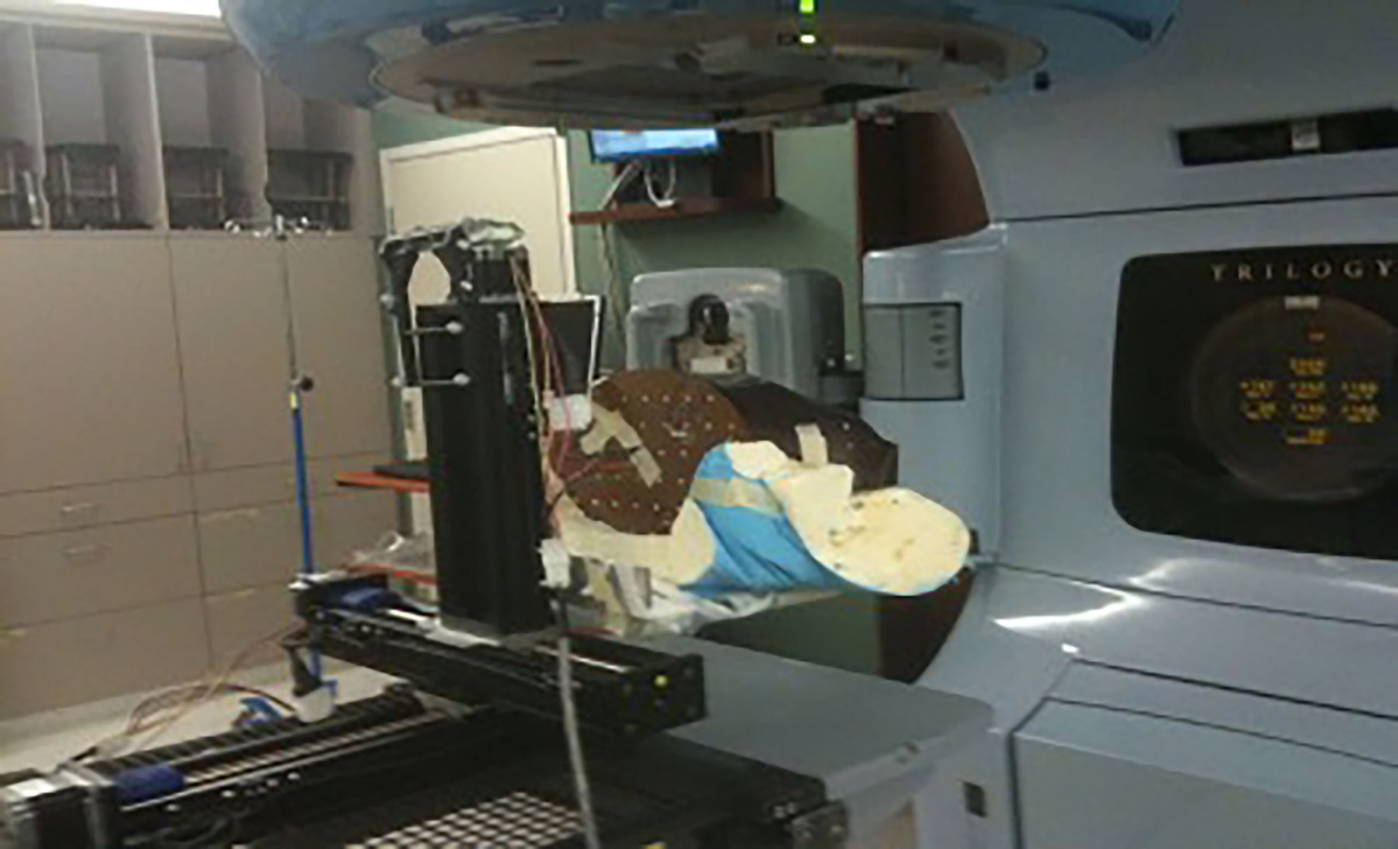
Experimental setup showing the RANDO phantom immobilized with alpha-cradle on a motion platform.

**Figure 2:**
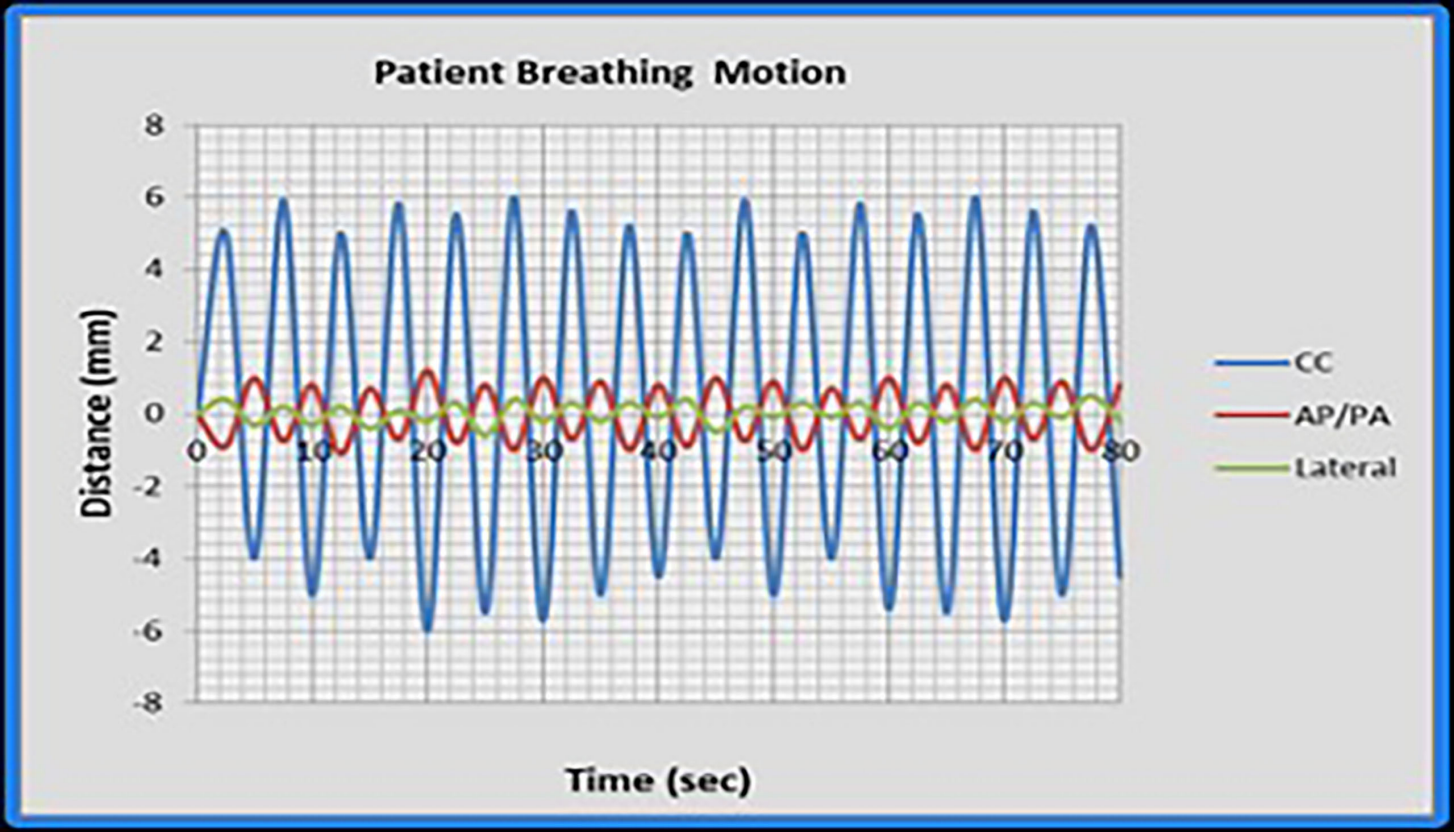
The respiratory motion M3 as simulated in this study. Cranio-caudal (CC); anteroposterior (AP); and lateral (LAT) directions.

An expanding foam structure was created to support the phantom and provide repeatable positioning. The motion profiles were generated with an in-house programmable motion platform as shown in **Figure 1**. Only the chest sub-plan was used in this study as the aim of this study is to evaluate the impact of respiratory motion on the lung dose. The RANDO phantom was first set up to the predetermined isocenter location using surface marks and lasers and then the chest sub-plan treatment was delivered using a Varian Trilogy linear accelerator (Varian Medical Systems Inc., Palo Alto, CA). Dose measurement was first carried out in a static (no motion) phantom as a reference and was repeated with the phantom in motion. We used typical published peak-to-peak motion amplitudes: 5, 8, and 12 mm in craniocaudal (CC) direction for M1, M2, and M3, respectively, and 2 mm for both AP and LAT directions (26, 30, 31). **Figure 2** displays the respiratory motion for M3. The motion platform was set in motion and the treatment dose delivery was started after a random delay, as would happen for a patient in the clinic. Additional measurements were carried out with 5-, 8-, and 12-mm peak-to-peak amplitudes in the CC direction only to evaluate the impact of directional respiratory motion during IMTMI.

### Statistical analysis

Two-sided, paired Student’s *t*-test evaluated statistical significance with *p*-values < 0.05 using GraphPad Instat version 3.05 (GraphPad Software, San Diego, CA, USA).

### Patient QA measurement with ArcCHECK

**Figure 3:**
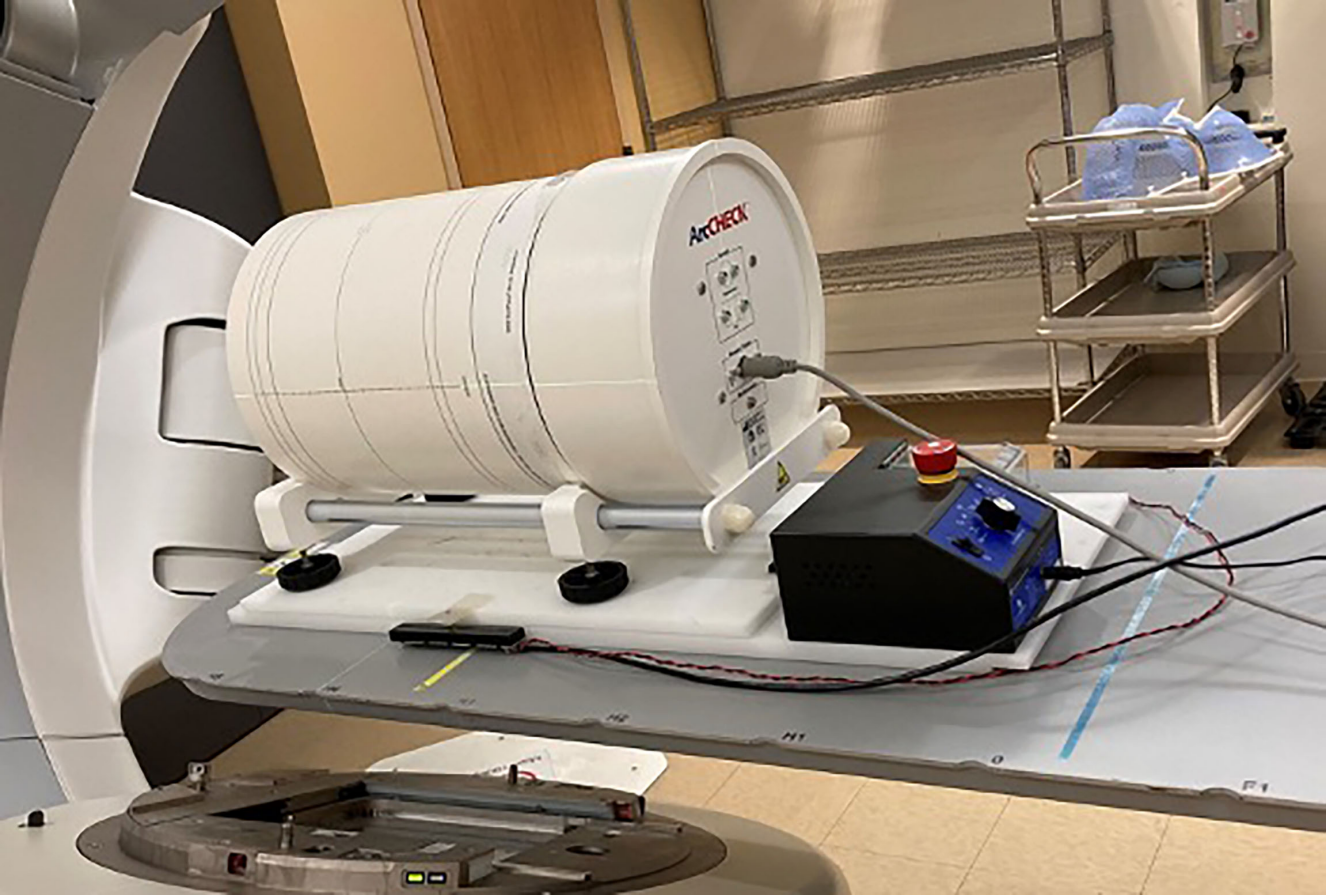
ArcCHECK detector placed array on the motion platform before dose measurement.

Patient-specific QA for routine IMRT and VMAT in our clinic is done with ArcCHECK (Sun Nuclear, Melbourne, FL), a 3D cylindrical phantom with a diameter of 21 cm and a helical detector grid consisting of 1,386 diode detectors (0.8 × 0.8 mm^2^) placed at intervals of 10 mm. We placed the ArcCHECK on a commercial motion platform as shown in **Figure 3** and repeated the treatment delivery and measurement for plans from 5 patients who were treated in our clinic with 9 Gy (150 cGy BID) IMTMI while simulating an extreme case of respiratory motion with a 15 mm excursion in only the CC direction. Gamma index analysis was performed and compared with and without motion using 3%, 5%, and 10% dose agreement with a 3-mm constant distance to agreement (DTA).

### 3DVH dose reconstruction

The ArcCHECK-3DVH system (Sun Nuclear Corporation, Melbourne, FL, USA) is a commercial DVH-based QA tool. The 3D patient dose is constructed from the measurement data with the provided internal calculation engine, called ArcCHECK planned dose perturbation (ACPDP). The ACPDP algorithm involves the following calculation steps: (a) synchronizing the planned data with the ArcCHECK virtual inclinometer recorded data; (b) generating a relative 3D dose grid to a homogeneous cylindrical phantom for each sub-beam; (c) morphing the relative dose based on the ArcCHECK-measured data to produce the 3D absolute dose in the cylindrical phantom; (d) taking the ratio of the reconstructed dose to the TPS-calculated dose for each voxel in the phantom; and (e) perturbing the TPS-calculated dose of the patient by the above ratios (36). The final grid size of the reconstructed dose was kept the same as that of the TPS dose calculation. To perform 3DVH reconstruction, the following data set was gathered: (1) reference DICOM RT plan, (2) DICOM RT dose (TPS-calculated dose for the patient and ArcCHECK geometries, respectively), and (3) ArcCHECK measurement data (.acml).

## Results

### Point dose measurements with TLD

**Figure 4:**
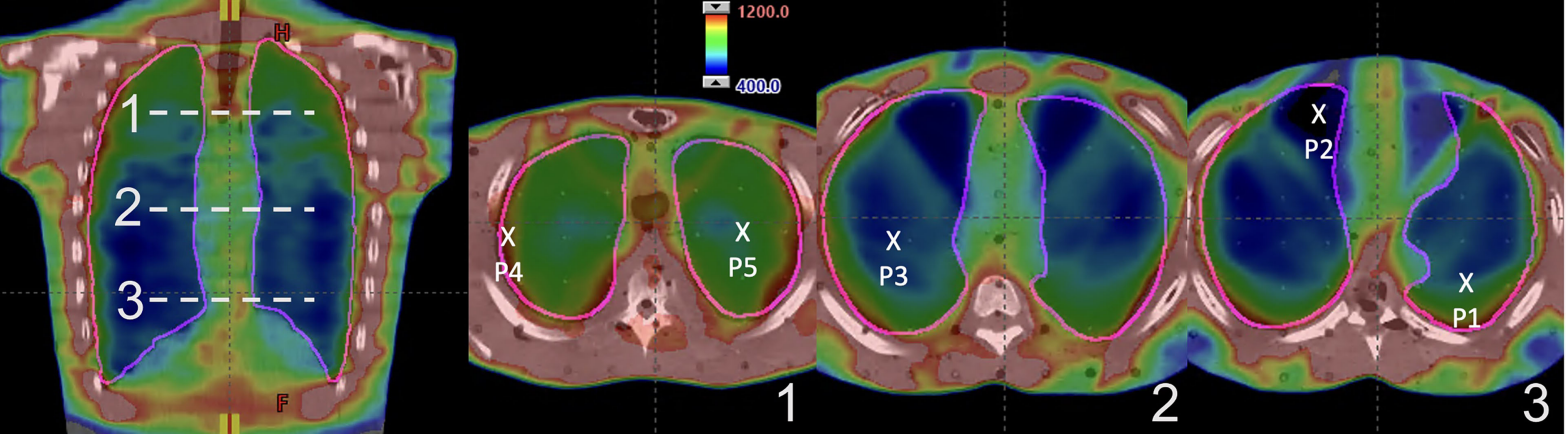
IMTMI dose distribution in the RANDO phantom. A coronal slice on the left with the indexing (1–3) for the axial slices where the TLDs were placed. On the right are the three axial slices showing the five measurement points within the lung. Dose range is shown from 400 cGy (blue) to 1,200 cGy (red).

IMTMI dose distribution of the chest plan shown in **Figure 4** demonstrates the planned IMTMI dose coverage for the PTV in the chest and sparing of the lungs in the RANDO phantom. Dose ranged from 4 Gy (blue) to 12 Gy (red). A sharp reduction beyond the target was achieved, which provided a lower dose to surrounding healthy tissue. Dose in the coronal view shown in the left pane also displays the index for three axial planes where the TLD measurements were done. On the right, the three axial planes displayed dose distribution and the location of five measurement points.

**Figure 5:**
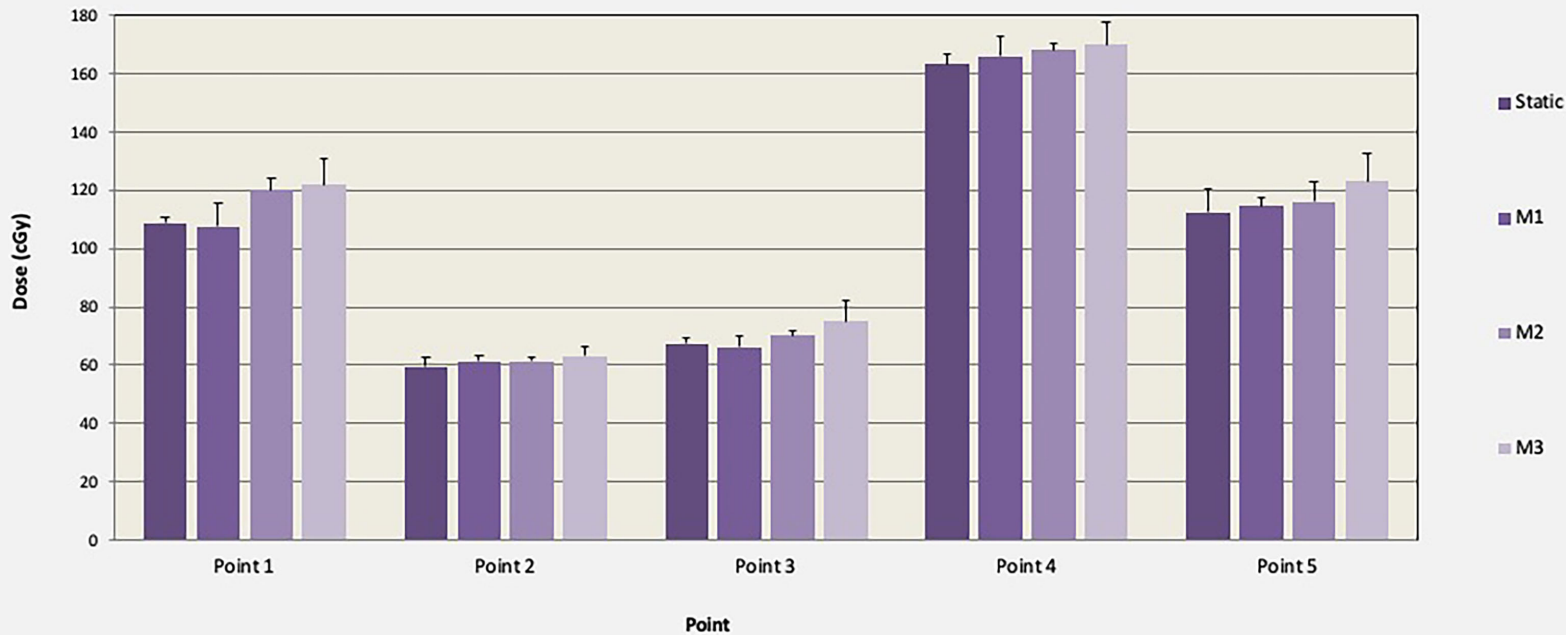
Comparison of TLD dose and associated standard deviations (error bars) in cGy with and without motion (M1, M2, and M3) for one TMI fraction of chest plan (150 cGy).

TLD measured doses and associated standard deviations (error bars) in five points within the lungs are shown in **Figure 5** for the motions M1, M2, and M3. TLD measurements were normalized to the static reference dose obtained irradiation with no motion. The mean normalized TLD readings (range) were 1.01 (0.98–1.03), 1.04 (1.01–1.09), and 1.08 (1.04–1.12) for M1, M2, and M3, respectively. A statistically significant change in delivered dose was observed for M2 and M3 (*p* < 0.05). Additional measurements performed with 5-, 8-, and 12-mm motion in CC direction only agreed within 1% with the respiratory motions M1, M2, and M3, indicating that the impact of respiratory motion in LAT and AP directions may negligible during IMTMI.

### Treatment delivery verification with ArcCHECK

**Figure 6:**
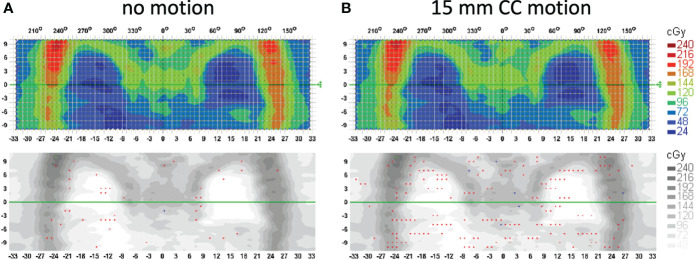
Treatment delivery QA comparison for a patient with **(A)** no motion and **(B)** 15 mm CC motion. Measurements were done with an ArcCHECK detector array and analysis is performed with the gamma index criteria of 3%/3 mm. Red and blue dots show the locations (detectors) that fail the 3%/3 mm gamma index passing criteria.

**Figure 7:**
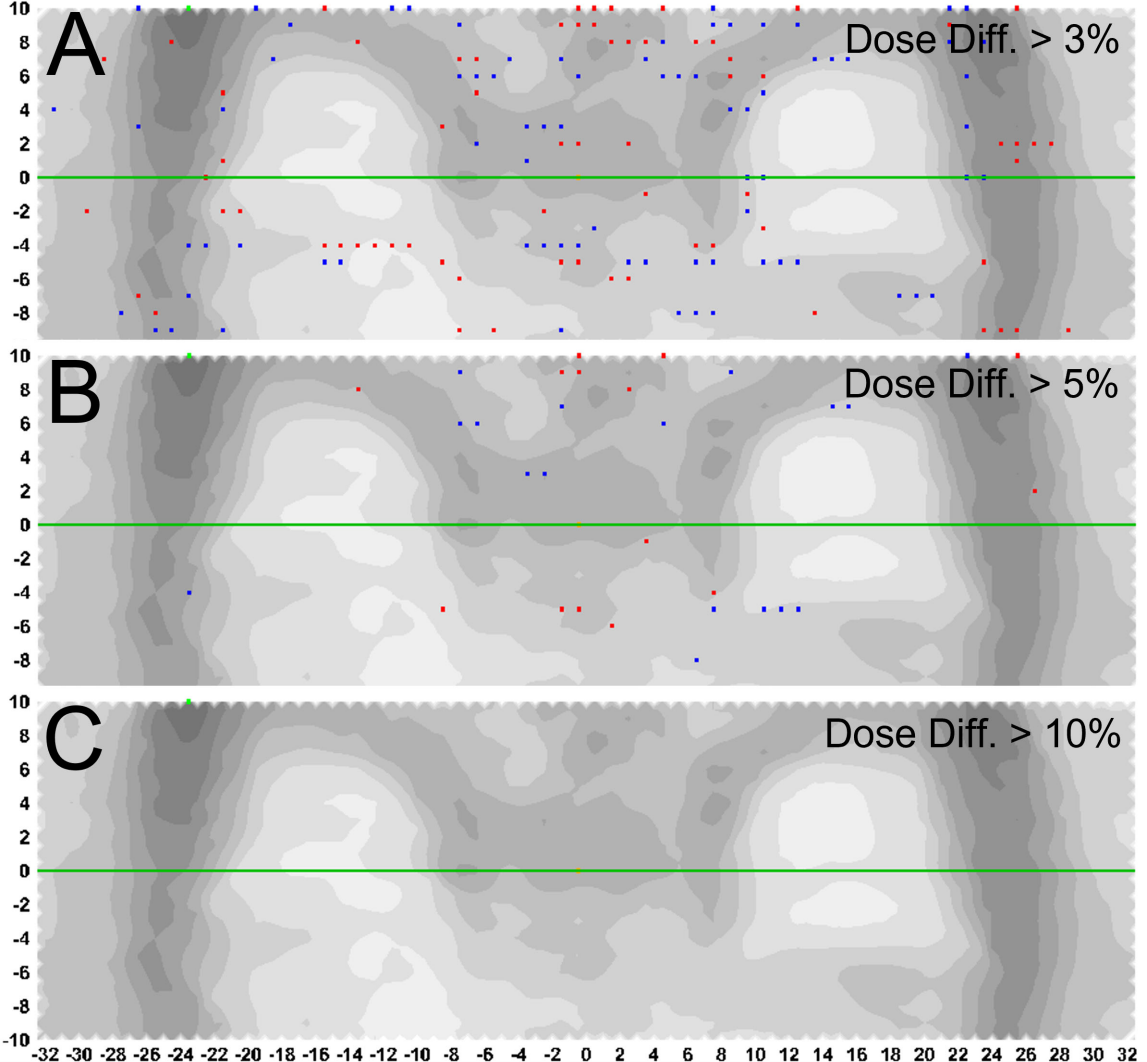
Percent difference in measured dose with and without motion. Blue (+) and red (-) dots represent the detectors or location within the lungs with a measured difference greater than **(A)** 3%, **(B)** 5%, and **(C)** 10%, with 3mm distance to agreement criteria.

**Figure 8:**
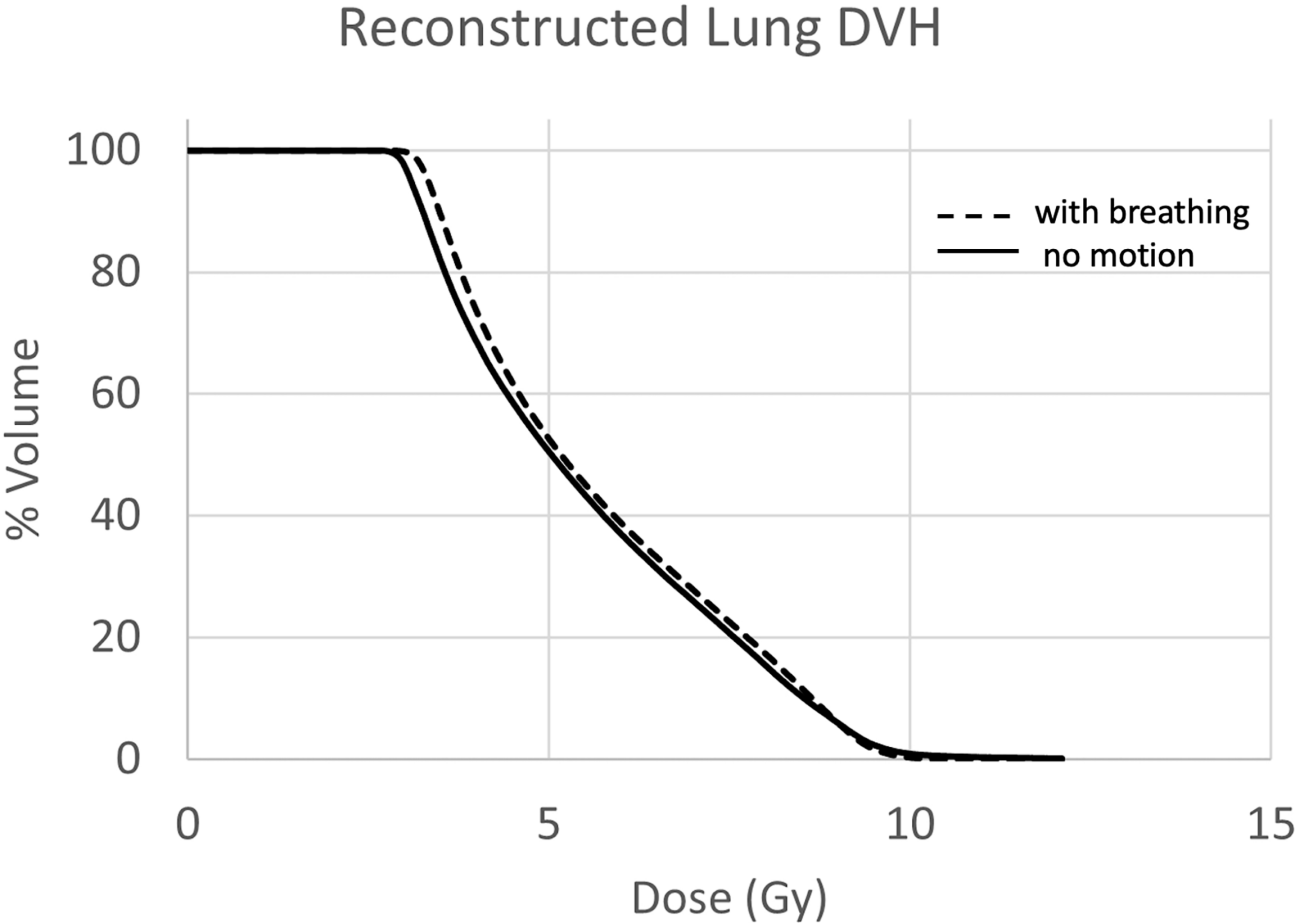
Lung DVH comparison with (solid) and without (dashed) motion. The mean dose difference was less than 3%.

Treatment delivery dose map comparison obtained with and without motion using ArcCHECK for a representative patient is shown in **Figure 6**. The IMTMI. chest sub-plan had a 96.8% GPR with no motion and 87.1% with motion with 3%/3 mm criteria. This indicates that respiratory motion caused an additional 10.3% of the detectors to measure a dose difference greater than 3%. When the dose difference criterion was increased to 5% with a constant 3-mm DTA, the GPR differed by 2.8% (97% vs. 99.8%). **Figure 7** compares the measured dose differences for the three-dose agreement levels used in this study: 3%, 5%, and 10%. Both the blue (+) and red (−) dots identify the detectors or location within the lungs with a measured difference of more than the specified level with motion. As the dose difference criteria increased from 3% to 10%, the number of detectors detecting such a dose difference decreased from 134 to only 1 in 1,386 detectors, respectively. This indicates that the motion would change the dose by more than 10% only in one small, isolated location within the lung of the same patient during IMTMI. For the cohort of 5 patients, the average percent differences in GPR due to respiratory motion was 11.1 ± 2.1% with 3%/3 mm. Nonetheless, it was only 3.8 ± 1.5%, and 0.1 ± 0.2% when a dose agreement criterion of 5% and 10% was used, respectively. **Figure 8** shows the comparison of 3DVH for the same patient with and without motion. The percent difference in mean lung dose was less than 3% with motion. For the cohort of five patients evaluated in this study, the effect of respiratory motion on the mean lung dose (5.7 ± 0.3 Gy vs. 5.5 ± 0.2 Gy) was not statistically significant based on the Student’s *t*-test (*p* > 0.05).

## Discussion

Organ motion is by far the largest contributor to uncertainties in RT. Respiratory motion affects all tumor sites in the thorax and abdomen and is the most profound and relevant for radiotherapy. Organ motion, dose uncertainty, motion mitigation, and management strategies in lung cancer have been studied extensively. Previous IMRT studies have indicated increasing dose discrepancies ranging from 3% to 12% between planned and delivered doses (37–40) due to respiratory motion. Treatment delivery with higher dose rates and smaller monitor-unit (MU) per segment has been associated with larger dosimetric errors (41, 42). Seco et al. (43) argued that interplay between organ (breathing) motion and leaf motion is only significant when considering the case of treatment beams made up of many few-monitor-unit segments, where the segment delivery time (1–2 s) is of the order of the respiratory period (3–5 s). During IMRT with small numbers of MUs per segment, the difference between the motion-averaged and static dose for 30 fractions could range from 6% to 12% for simple to complex respiratory motion functions, respectively (44).

TMI being a complex treatment technique delivering small numbers of MUs per leaf segment is prone to large delivery uncertainties especially when treating bones in the chest and maximally sparing lungs at the same time. Several studies investigated the dosimetric accuracy of both helical and linac-based TMI delivery techniques in human-like phantoms and confirmed that TMI is, regardless of the delivery technique, dosimetrically accurate and safe (5, 17, 44). These studies, nonetheless, were conducted in the “ideal” situation without intrafraction motion. In this study, we performed a comprehensive investigation of dose uncertainty in lungs due to respiratory motion during linac-based IMTMI delivery. To achieve this, an end-to-end test was carried out through immobilization, simulation, planning, treatment delivery, and dose measurement with and without motion using an anthropomorphic phantom and an ArcCHECK placed on a motion platform. When an extreme case of respiratory motion was simulated with a 15-mm peak-to-peak displacement only the CC direction during IMTMI, none of the patients had more than 6 out of 1386 (0.4%) detectors reporting more than a 10% dose difference. Similarly, we observed a dose difference of more than 5% in only 53 ± 21 (3.8 ± 1.5%) detectors. These results indicate that respiratory motions increase the dose only in small and isolated parts within the lungs. Moreover, the mean lung dose, which is the most relevant measure for toxicity, was not impacted by respiratory motion. One possible explanation for this observation is that the longer treatment times during TMI could have a dose averaging effect. Considering the average lung motion amplitude is 6.0 ± 2.2 in approximately 90% of the patients with a maximum of 12 ± 2 mm (29, 30), it may be safe to assume that an overwhelming majority of patients treated with TMI will have a negligible dose uncertainty due to respiratory motion.

One of the potential limitations of this study is that the respiratory motion was applied to the whole phantom. Nonetheless, our approach is adequate to study the impact of respiratory motion on the lung dose and ignores the dose uncertainty in the target (bony anatomy). Bones in our body are not affected by respiratory motion except for the ribcage, which constitutes only a small portion of the target in TMI. However, further analysis could include more realistic motions to be simulated separately for targets and lungs.

Initial clinical trials have demonstrated that the TMI-inclusive transplant regimens are safe and feasible (22–24, 45). Several Phase 2 studies are ongoing to establish the outcome benefit of adding TMI to the current standard of care (46–48). Furthermore, there is an increased interest in dose escalation studies based on the reports that a TBI dose of 15.6 Gy (30% more than the standard dose of 12 Gy) halved the relapse rate (8). However, treatment outcomes did not improve due to radiation toxicity (9). Respiratory motion is a concern in the management of radiation toxicity as it has the potential to increase the mean lung dose. Our study suggests that the impact of respiratory motion on the lung dose may be negligible. This is particularly assuring as there may be a therapeutic benefit of higher TMI doses, especially for patients with advanced hematological malignancies with poor prognoses.

## Data availability statement

The datasets presented in this article are not readily available. Requests to access the datasets should be directed to baydogan@uchicago.edu.

## Author contributions

BA and AGK responsible for the conception and design. BA provided oversight as the senior author. AGK, MS, KHA, and BA conducted the experiments. AGK performed statistical analyses. AGK, MS, KHA, EP and BA interpreted the experimental results. EP provided scientific review and improvements. AGK drafted the manuscript. All authors revised and approved the manuscript for submission.

## Acknowledgments

The authors thank Dr. Rodney Wiersma for providing his in-house 3D motion platform to perform the TLD measurements in this study. A portion of the study was presented at the AAPM annual meeting in 2011.

## Conflict of interest

BA received grant support from Varian Medical Systems Inc, Palo Alto CA.

The remaining authors declare that the research was conducted in the absence of any commercial or financial relationships that could be construed as a potential conflict of interest.

## Publisher’s note

All claims expressed in this article are solely those of the authors and do not necessarily represent those of their affiliated organizations, or those of the publisher, the editors and the reviewers. Any product that may be evaluated in this article, or claim that may be made by its manufacturer, is not guaranteed or endorsed by the publisher.

## References

[1] Sanders JE. Late effects in children receiving total body irradiation for bone marrow transplantation. Radiother Oncol (1990) 18 Suppl 1:82–7. doi: 10.1016/0167-8140(90)90181-U 2247652

[2] Brochstein JA Kernan NA Groshen S Cirrincione C Shank B Emanuel D. Allogeneic bone marrow transplantation after hyperfractionated total-body irradiation and cyclophosphamide in children with acute leukemia. N Engl J Med (1987) 317(26):1618–24. doi: 10.1056/NEJM198712243172602 3317056

[3] Wong JY Filippi AR Dabaja BS Yahalom J Specht L. Total body irradiation: Guidelines from the international lymphoma radiation oncology group (ILROG). Int J Radiat Oncol Biol Phys (2018) 101(3):521–9. doi: 10.1016/j.ijrobp.2018.04.071 29893272

[4] Wong JY Filippi AR Scorsetti M Hui S Muren LP Mancosu P. Total marrow and total lymphoid irradiation in bone marrow transplantation for acute leukemia. Lancet Oncol (2020) 21(10):e477–87. doi: 10.1016/S1470-2045(20)30342-9 33002443

[5] Hui SK Das RK Thomadsen B Henderson D. CT-based analysis of dose homogeneity in total body irradiation using lateral beam. J Appl Clin Med Phys (2004) 5:71–9. doi: 10.1120/jacmp.v5i4.1980 PMC5723515 15738922

[6] Hui SK Verneris MR Froelich J Dusenbery K Welsh JS. Multimodality image guided total marrow irradiation and verification of the dose delivered to the lung, PTV, and thoracic bone in a patient: a case study. Technol Cancer Res Treat (2009) 8(1):23–8. doi: 10.1177/153303460900800104 19166239

[7] Carruthers SA Wallington MM. Total body irradiation and pneumonitis risk: a review of outcomes. Br J Cancer (2004) 90(11):2080–4. doi: 10.1038/sj.bjc.6601751 PMC2409505 15150598

[8] Clift RA Buckner CD Appelbaum FR Bearman SI Petersen FB Fisher LD. Allogeneic marrow transplantation in patients with acute myeloid leukemia in first remission: a randomized trial of two irradiation regimens. Blood (1990) 76(9):1867–71. doi: 10.1182/blood.V76.9.1867.1867 2224134

[9] Clift RA Buckner CD Appelbaum FR Bryant E Bearman SI Petersen FB. Allogeneic marrow transplantation in patients with chronic myeloid leukemia in the chronic phase: a randomized trial of two irradiation regimens. Blood (1991) 77(8):1660–5. doi: 10.1182/blood.V77.8.1660.1660 2015394

[10] Ozsahin M Pène F Touboul E Gindrey-Vie B Dominique C Lefkopoulos D. Total-body irradiation before bone marrow transplantation. results of two randomized instantaneous dose rates in 157 patients. Cancer (1992) 69(11):2853–65. doi: 10.1002/1097-0142(19920601)69:11<2853::aid-cncr2820691135>3.0.co;2-2 1571917

[11] Dusenbery KE Daniels KA McClure JS McGlave PB Ramsay NK Blazar BR. Randomized comparison of cyclophosphamide-total body irradiation versus busulfan-cyclophosphamide conditioning in autologous bone marrow transplantation for acute myeloid leukemia. Int J Radiat Oncol Biol Phys (1995) 31(1):119–28. doi: 10.1016/0360-3016(94)00335-I 7995742

[12] Devergie A Blaise D Attal M Tigaud JD Jouet JP Vernant JP. Allogeneic bone marrow transplantation for chronic myeloid leukemia in first chronic phase: a randomized trial of busulfan-cytoxan versus cytoxan-total body irradiation as preparative regimen: a report from the French society of bone marrow graft (SFGM). Blood (1995) 85(8):2263–8. doi: 10.1182/blood.V85.8.2263.bloodjournal8582263 7718899

[13] Della Volpe A Ferreri AJ Annaloro C Mangili P Rosso A Calandrino R. Lethal pulmonary complications significantly correlate with individually assessed mean lung dose in patients with hematologic malignancies treated with total body irradiation. Int J Radiat Oncol Biol Phys (2002) 52(2):483–8. doi: 10.1016/S0360-3016(01)02589-5PMID:11872296 11872296

[14] Hui SK Kapatoes J Fowler J Henderson D Olivera G Manon RR. Feasibility study of helical tomotherapy for total body or total marrow irradiation. Med Phys (2005) 32(10):3214–24. doi: 10.1118/1.2044428 16279075

[15] Aydogan B Mundt AJ Roeske JC. Linac-based intensity modulated total marrow irradiation (IM-TMI). Technol Cancer Res Treat (2006) 5(5):513–9. doi: 10.1177/153303460600500508 16981794

[16] Wong JY Liu A Schultheiss T Popplewell L Stein A Rosenthal J. Targeted total marrow irradiation using three-dimensional image-guided tomographic intensity-modulated radiation therapy: an alternative to standard total body irradiation. Biol Blood Marrow Transplant (2006) 12(3):306–15. doi: 10.1016/j.bbmt.2005.10.026 16503500

[17] Wilkie JR Tiryaki H Smith BD Roeske JC Radosevich JA Aydogan B. Feasibility study for linac-based intensity modulated total marrow irradiation. Med Phys (2008) 35(12):5609–18. doi: 10.1118/1.2990779 19175118

[18] Yeginer M Roeske JC Radosevich JA Aydogan B. Linear accelerator-based intensity-modulated total marrow irradiation technique for treatment of hematologic malignancies: a dosimetric feasibility study. Int J Radiat Oncol Biol Phys (2011) 79(4):1256–65. doi: 10.1016/j.ijrobp.2010.06.029 21035960

[19] Mancosu P Cozzi L Muren LP. Total marrow irradiation for hematopoietic malignancies using volumetric modulated arc therapy: A review of treatment planning studies. Phys Imaging Radiat Oncol (2019) 11:47–53. doi: 10.1016/j.phro.2019.08.001 33458277 PMC7807866

[20] Aydogan B Yeginer M Kavak GO Fan J Radosevich JA Gwe-Ya K. Total marrow irradiation with RapidArc volumetric arc therapy. Int J Radiat Oncol Biol Phys (2011) 81(2):592–9. doi: 10.1016/j.ijrobp.2010.11.035 21345619

[21] Fogliata A Cozzi L Clivio A Ibatici A Mancosu P Navarria P. Preclinical assessment of volumetric modulated arc therapy for total marrow irradiation. Int J Radiat Oncol Biol Phys (2011) 80(2):628–36. doi: 10.1016/j.ijrobp.2010.11.028 21277109

[22] Wong JY Forman S Somlo G Rosenthal J Liu A Schultheiss T. Dose escalation of total marrow irradiation with concurrent chemotherapy in patients with advanced acute leukemia undergoing allogeneic hematopoietic cell transplantation. Int J Radiat Oncol Biol Phys (2013) 85(1):148–56. doi: 10.1016/j.ijrobp.2012.03.033 PMC4312108 22592050

[23] Patel P Aydogan B Koshy M Mahmud D Oh A Saraf SL. Combination of linear accelerator-based intensity-modulated total marrow irradiation and myeloablative fludarabine/busulfan: a phase I study. Biol Blood Marrow Transplant (2014) 20(12):2034–4. doi: 10.1016/j.bbmt.2014.09.005 25234438

[24] Patel P Oh AL Koshy M Sweiss K Saraf SL Quigley JG. A phase 1 trial of autologous stem cell transplantation conditioned with melphalan 200 mg/m^2^ and total marrow irradiation (TMI) in patients with relapsed/refractory multiple myeloma. Leuk Lymphoma (2018) 59(7):1666–71. doi: 10.1080/10428194.2017.1390231 29065747

[25] George R Keall PJ Kini VR Vedam SS Siebers JV Wu Q. Quantifying the effect of intrafraction motion during breast IMRT planning and dose delivery. Med Phys (2003) 30(4):552–62. doi: 10.1118/1.1543151 12722807

[26] Quirk S Becker N Smith WL. External respiratory motion analysis and statistics for patients and volunteers. J Appl Clin Med Phys (2013) 14(2):4051. doi: 10.1120/jacmp.v14i2.4051 23470934 PMC5714366

[27] Plathow C Fink C Ley S Puderbach M Eichinger M Zuna I. Measurement of tumor diameter-dependent mobility of lung tumors by dynamic MRI. Radiother Oncol (2004) 73(3):349–54. doi: 10.1016/j.radonc.2004.07.017 15588881

[28] Keall PJ Mageras GS Balter JM Emery RS Forster KM Jiang SB. The management of respiratory motion in radiation oncology report of AAPM task group 76. Med Phys (2006) 33(10):3874–900. doi: 10.1118/1.2349696 17089851

[29] Knybel L Cvek J Molenda L Stieberova N Feltl D. Analysis of lung tumor motion in a Large sample: Patterns and factors influencing precise delineation of internal target volume. Int J Radiat Oncol Biol Phys (2016) 96(4):751–8. doi: 10.1016/j.ijrobp.2016.08.008 27788948

[30] Liu HH Balter P Tutt T Choi B Zhang J Wang C. Assessing respiration-induced tumor motion and internal target volume using four-dimensional computed tomography for radiotherapy of lung cancer. Int J Radiat Oncol Biol Phys (2007) 68(2):531–40. doi: 10.1016/j.ijrobp.2006.12.066 17398035

[31] Seppenwoolde Y Shirato H Kitamura K Shimizu S van Herk M Lebesque JV. Precise and real-time measurement of 3D tumor motion in lung due to respiratory and heartbeat, measured during radiotherapy. Int J Radiat Oncol Biol Phys (2002) 53(4):822–34. doi: 10.1016/S0360-3016(02)02803-1 12095547

[32] Zelefsky MJ Fuks Z Happersett L Lee HJ Ling CC Burman CM. Clinical experience with intensity modulated radiation therapy (IMRT) in prostate cancer. Radiother Oncol (2000) 55(3):241–9. doi: 10.1016/S0167-8140(99)00100-0 10869739

[33] Xing L Lin Z Donaldson SS Le QT Tate D Goffinet DR. Dosimetric effects of patient displacement and collimator and gantry angle misalignment on intensity modulated radiation therapy. Radiother Oncol (2000) 56(1):97–108. doi: 10.1016/S0167-8140(00)00192-4 10869760

[34] Manning MA Wu Q Cardinale RM Mohan R Lauve AD Kavanagh BD. The effect of setup uncertainty on normal tissue sparing with IMRT for head-and-neck cancer. Int J Radiat Oncol Biol Phys (2001) 51(5):1400–9. doi: 10.1016/S0360-3016(01)01740-0 11728701

[35] Reft CS Runkel-Muller R Myrianthopoulos L. *In vivo* and phantom measurements of the secondary photon and neutron doses for prostate patients undergoing 18 MV IMRT. Med Phys (2006) 33(10):3734–42. doi: 10.1118/1.2349699 17089839

[36] Saito M Kadoya N Sato K Ito K Dobashi S Takeda K. Comparison of DVH-based plan verification methods for VMAT: ArcCHECK-3DVH system and dynalog-based dose reconstruction. J Appl Clin Med Phys (2017) 18(4):206–14. doi: 10.1002/acm2.12123 PMC5875843 28649722

[37] Jiang SB Pope C Al Jarrah KM Kung JH Bortfeld T Chen GT. An experimental investigation on intra-fractional organ motion effects in lung IMRT treatments. Phys Med Biol (2003) 48(12):1773–84. doi: 10.1088/0031-9155/48/12/307 12870582

[38] Yu CX Jaffray DA Wong JW. The effects of intra-fraction organ motion on the delivery of dynamic intensity modulation. Phys Med Biol (1998) 43(1):91–104. doi: 10.1088/0031-9155/43/1/006 9483625

[39] Zhao B Yang Y Li T Li X Heron DE Huq MS. Statistical analysis of target motion in gated lung stereotactic body radiation therapy. Phys Med Biol (2011) 56(5):1385–95. doi: 10.1088/0031-9155/56/5/011 21317481

[40] Zhao B Yang Y Li T Li X Heron DE Huq MS. Dosimetric effect of intrafraction tumor motion in phase gated lung stereotactic body radiotherapy. Med Phys (2012) 39(11):6629–37. doi: 10.1118/1.4757916 23127057

[41] Li H Park P Liu W Matney J Liao Z Balter P. Patient-specific quantification of respiratory motion-induced dose uncertainty for step-and-shoot IMRT of lung cancer. Med Phys (2013) 40(12):121712. doi: 10.1118/1.4829522 24320498 PMC3843761

[42] Berbeco RI Pope CJ Jiang SB. Measurement of the interplay effect in lung IMRT treatment using EDR2 films. J Appl Clin Med Phys (2006) 7(4):33–42. doi: 10.1120/jacmp.v7i4.2222 17533350 PMC5722391

[43] Seco J Sharp GC Turcotte J Gierga D Bortfeld T Paganetti H. Effects of organ motion on IMRT treatments with segments of few monitor units. Med Phys (2007) 34(3):923–34. doi: 10.1118/1.2436972 PMC2034283 17441238

[44] Surucu M Yeginer M Kavak GO Fan J Radosevich JA Aydogan B. Verification of dose distribution for volumetric modulated arc therapy total marrow irradiation in a humanlike phantom. Med Phys (2012) 39(1):281–8. doi: 10.1118/1.3668055 22225298

[45] Tran MC Hasan Y Wang AY. A phase 1 trial utilizing TMI with fludarabine-melphalan in patients with hematologic malignancies undergoing second allo-SCT. Blood Adv (2022) bloodadvances.2022007530. doi: 10.1182/bloodadvances.2022007530 PMC9898602 35851593

[46] Wong JY Tsai NC Han C Palmer J Liu A Al Malki M. Phase II study of dose escalated total marrow and lymphoid irradiation (TMLI) in combination with cyclophosphamide and etoposide in patients with poor-risk acute leukemia. Int J Radiat Oncol Biol Phys (2020) 108(3):S155. doi: 10.1016/j.ijrobp.2020.07.912

[47] Available at: https://www.clinicaltrials.gov/ct2/show/NCT03121014.

[48] Available at: https://clinicaltrials.gov/ct2/show/NCT04187105.

